# The Experiences of Adults Experiencing Homelessness When Accessing and Using Psychosocial Interventions: A Systematic Review and Qualitative Evidence Synthesis

**DOI:** 10.1002/cl2.70036

**Published:** 2025-04-03

**Authors:** Chris O'Leary, Esther Coren, Anton Roberts

**Affiliations:** ^1^ Policy Evaluation and Research Unit Manchester Metropolitan University Manchester UK; ^2^ School of Public Health, Midwifery and Social Work, Sidney De Haan Research Centre for Arts and Health Canterbury Christ Church University Canterbury UK

**Keywords:** experiences, homelessness, psychosocial interventions, thematic synthesis

## Abstract

**Background:**

Adults experiencing homelessness in high‐income countries are more likely to have mental ill‐health and engage in problematic substance use. They are also more likely to experience challenges when accessing services. Psychosocial interventions are increasingly used with this group. Most of the evidence around these interventions is not specific to their use with adults experiencing homelessness.

**Objectives:**

To summarise the best available evidence of the views and experiences of adults experiencing homelessness in high‐income countries about psychosocial interventions.

**Search Methods:**

This review is based on evidence identified in an Evidence and Gap Map (EGM) on interventions for people experiencing homelessness. The EGM searches were conducted in September 2021. Additionally, we undertook a call for evidence and hand searches of key journals.

**Selection Criteria:**

We included qualitative data from studies of psychosocial interventions. Participants were adults aged 18+ experiencing homelessness in high‐income countries. Only studies that reported the views, opinions, perceptions, and experiences of participants were included.

**Data Collection and Analysis:**

Of the 468 studies originally screened, 17 were eligible for full‐text review, which was undertaken independently by two reviewers. Ten were excluded at this stage, and seven were identified as meeting the inclusion criteria. Analysis was undertaken using thematic synthesis in three stages: (1) findings data were extracted from studies. Two reviewers independently extracted findings from included studies. These were compared and agreed on which findings to include for analysis; (2) two reviewers gave each line of extracted data a descriptive code (a short descriptive summary). These were compared and a set of codes for inclusion in the next stage of analysis was agreed; (3) the reviewers iteratively examined the descriptive themes, inferring from these themes the experiences of participants and their perceptions of how the intervention worked for them. These analytical themes were discussed with a panel of people with experience of homelessness.

**Main Results:**

Seven studies were included in this review, covering several intervention types. A total of 84 adults experiencing homelessness were included in these studies. Three studies were conducted in Canada, three in the United States, and one in Scotland. All were published after 2009. The studies used various qualitative methods of data collection and analysis. None of the included studies were assessed as high quality. The most significant area of concern across the included studies concerned relationships between researchers and research participants, where five included studies were assessed as low quality. Areas of higher quality were clarity of research questions and methods. Overall, 368 lines of findings were extracted and coded under 118 descriptive codes. Of these, 55 related to direct quotes of participants' views and experiences. The remainder were the study authors' interpretations of the research participants' experiences. The 118 descriptive codes were grouped into 14 descriptive themes. The themes are descriptions of patterns in the data (the findings extracted from the included studies). These 14 descriptive themes (and the 118 descriptive codes underpinning them) summarise data from the primary studies. The final analysis stage was interpretation of the descriptive themes and development of analytical themes to answer the review questions. The reviewers were able to answer two of the four review questions: the experiences of participants when using psychosocial interventions, and whether they felt the interventions worked for them. The question concerning underlying theories of how the interventions are intended to work was addressed through a separate analysis. The question of differences between interventions could not be answered because of the small number of included studies. The final analysis stage identified three analytical themes. These are: (1) the individual plays a pivotal role in their recovery and change journey; (2) accessibility is a key component of intervention success; and (3) relationships are an important intervention ingredient.

**Author's Conclusions:**

The reviewers draw two broad conclusions from this analysis: (1) it is important to place adults experiencing homelessness at the centre of the design of psychosocial interventions; and (2) it is important to treat adults experiencing homelessness as individuals.

## 1 Plain Language Summary

Qualitative synthesis finds that psychological interventions for people experiencing homelessness may benefit from an individualised approach, focus on relationships and accessibility.

### 1.1 The Review in Brief

Evidence from 7 studies with views and experiences of 84 adults experiencing homelessness suggest that individual goals and motivation, relationships with staff and peers, and the accessibility of services, are important factors.

### 1.2 What Is This Review About?

Adults experiencing homelessness are more likely than the general population to experience mental ill‐health and problematic substance use. They are also more likely to face challenges when accessing services to address these issues.

Psychosocial interventions focus on an individual's psychological development and their engagement with society and the community. They promote change in behaviour or thinking, and intend that these changes lead to reduced substance use, more stable housing, or improved mental health.

This review looked at the views and perceptions of adults experiencing homelessness when they used psychosocial interventions. The aim was to see what challenges they faced when accessing services, and what factors supported intervention success.

### 1.3 What Is the Aim of This Review?

The aim of the review was to understand the experiences of adults experiencing homelessness when they access and use psychosocial interventions.

### 1.4 What Are the Main Findings of This Review?

The review team identified and considered 96 studies that might be relevant, and finally included 7 studies in the analysis. The studies covered various different interventions. A total of 84 adults experiencing homelessness were included in the 7 included studies. There was an even mix of women and men. Three studies were conducted in Canada, three in the United States, and one in Scotland. All were published after 2009. The studies used a variety of data collection and analysis methods that capture the views and experiences of people using the interventions.

None of the included studies were assessed as high quality. The most significant area of concern across the included studies concerned relationships between researchers and research participants, where five included studies were assessed as low quality. Areas of higher quality were clarity of the research questions and methods.

This review used ‘thematic synthesis’, finding themes across the studies to analyse and draw conclusions. This is a three‐stage process of extracting, analysing, and summarising study findings. Each line (sentence) was given a descriptive code. There were 118 total codes, of which 55 were direct quotes from adults experiencing homelessness. These codes were then grouped into 14 ‘descriptive themes’. The final analysis stage grouped the 14 descriptive themes into three overall (analytic) themes: (1) the individual plays a key role in their recovery and change journey; (2) accessibility is a key component of intervention success; (3) relationships are an important intervention ingredient.

### 1.5 What Do the Findings of This Review Mean?

There are three implications identified in this review. First, the hope, motivation, and goals of individuals using these interventions could be an important ingredient, but most research does not consider this. More research is needed to understand whether these are important success factors. Second, drawing on this review and a companion review on the effectiveness of psychosocial interventions, there is no direct connection between the interventions covered by the two types of study. This lack of complementarity limits the explanatory potential across both types of study and their contribution to knowledge and policy. Finally, there is a limited geographic range of relevant studies. More research is needed outside North America.

### 1.6 How Up‐to‐Date Is This Review?

The review search was conducted in September 2021. Subject to funding, the authors aim to update this review in the future.

## 2 Background

### 2.1 The Problem, Condition, or Issue

Homelessness is a significant and growing social issue in many high‐income countries (Toro 2007). For example, in the United States, the Department for Housing and Urban Development reported that 582,500 people were experiencing homelessness in early 2022 (de Sousa et al. 2022). In Canada, around 35,000 people experience homelessness each night, and between 250,000 and 300,000 experience homelessness each year (Gaetz et al. 2016, as cited in Wong et al. 2020). Homelessness continues to rise in most European Union (EU) countries, with some 700,000 people experiencing homelessness in the EU in 2019 (FEANTSA 2022). In England, all forms of homelessness rose between 2008 and 2017 (O'Leary and Simcock 2020). According to the most up‐to‐date official statistics, almost 280,000 households were assessed as homeless or threatened with homelessness in 2021/22, and more than 3000 people were sleeping rough on a single night in England (DLUHC 2023).

Homelessness is a complex and multifaceted issue, with differences in how it is understood and experienced, and how these differences are conceptualised, described, and measured (O'Leary et al. 2022). Homelessness can be a traumatic experience, which can have a devastating effect on those experiencing it. Trauma can be a cause of someone's homelessness, and homelessness may be accompanied by further traumatic experiences. Several studies, some of which are cited below, have highlighted that more visible and extreme forms of homelessness (and particularly street homelessness) are often associated with adverse childhood events (Koh and Montgomery 2021), extreme social disadvantage (Mabhala et al. 2017), physical, emotional and sexual abuse (Green et al. 2012; Henny et al. 2007), neglect (Mar et al. 2014), low self‐esteem (Ravikumar‐Grant et al. 2023), poor physical and mental health (Vallesi et al. 2021), and much lower life expectancy compared to the general population (ONS 2019).

There is growing recognition by public policy‐makers that issues such as homelessness, drug and alcohol misuse, mental ill health, and offending may be experienced by the same people (Bramley et al. 2020). They often face what has been described as a ‘tri‐morbidity’ (Cornes et al. 2018); a combination of poor physical health, mental health, and problematic substance use (Cornes et al. 2018; Dobson 2019; Fitzpatrick et al. 2013).

Individuals experiencing homelessness have repeated, but intermittent, contact with a range of publicly funded services, particularly health (Aldridge et al. 2018), criminal justice (Bramley et al. 2020), and local government (Dobson 2019). Yet it is the case that many face significant barriers when they try to access mainstream public services (Armstrong et al. 2021; O'Leary et al. 2022). Barriers may arise because of the location and physical accessibility of services (Davies and Wood 2018), challenges in registering for services (Gunner et al. 2019), because they face stigma and discrimination (Gunner et al. 2019; Omerov et al. 2020; Reilly et al. 2022), lack of collaboration between services and the challenges this creates for people when coordinating contacts with multiple service providers (McNeill et al. 2022), and often fall through the cracks between different services they need to access (Dobson 2019). There is a substantive body of research that demonstrates that people experiencing homelessness face significant barriers accessing primary healthcare services, and may rely on acute and emergency services (Siersbaek et al. 2021).

One group of interventions that is increasingly used to address issues of mental ill‐health, substance use, and housing stability for people experiencing homelessness are psychosocial interventions. Although there is increasing evidence that these interventions work for the general population, there is more limited evidence that is specific to people experiencing homelessness. A sister review to the one set out here examines the effectiveness of psychosocial interventions for adults experiencing homelessness in relation to outcomes in mental ill health, substance use, and housing stability (O'Leary et al. 2022). However, given the substantive barriers that this population faces when trying to access public services (as evidenced above), it is also important to understand the experiences of adults experiencing homelessness when they access and use psychosocial interventions. It is this question that the review set out here sought to address (O'Leary et al. 2022).

### 2.2 The Intervention

#### 2.2.1 Defining Psychosocial Interventions

There is a lack of a single, agreed definition of psychosocial interventions (Hodges et al. 2011). One broad definition, and the one we use for this review, is provided by England et al. (2015) in their report ‘Psychosocial interventions for mental and substance use disorders: a framework for establishing evidence‐based standards’. This report was the outcome of detailed work by a committee of 16 experts established by the Institute of Medicine in the United States. In the report, Mary England and colleagues state that psychosocial interventions are ‘interpersonal or informational activities, techniques, or strategies that target biological, behavioral, cognitive, emotional, interpersonal, social, or environmental factors’ (England et al. 2015, 5) which aim to make positive changes to the lives of individuals engaging in these activities.

There are some commonalities underpinning the many definitions of what constitutes a psychosocial intervention. These include recognising that interventions have a change objective/aim, and that this intended change is psychological, and is often (though not exclusively) focused on mental health or substance use. Several include social change as well as psychological change as an objective, and all exclude interventions that are wholly or mostly pharmacological in approach. However, the extant literature also identifies huge variation in these interventions, including differences in setting, intensity, whether the intervention is group or individual based, and the treatment goals of the intervention. For this review, we propose to use the definition provided by England et al. (2015) and outlined above. This definition is relatively broad, and as such may not be helpful if assessing what constitutes and what does not constitute psychosocial interventions. We added further to this definition to focus on psychosocial interventions that are: (a) formally (though not necessarily universally) recognised as being psychosocial interventions; (b) are structured or planned, with an explicit intended goal or objective; (c) excludes pharmacological interventions (or interventions that are predominantly pharmacological in nature); and (d) targeted for use with adults experiencing homelessness. Our focus here is on psychosocial interventions that target individuals. Given this focus, we identified a list of 20 interventions that are the primary focus of this review, as set out in our protocol for this study (O'Leary et al. 2022; O'Leary et al. 2022). This typology is repeated in Table 1.

**Table 1:** Typology of interventions.

Category	Low intensity	High intensity
Talking therapy	Brief interventions	Motivational interviewing
	Brief motivational intervention	Motivational enhancement therapy
	Skills training	Cognitive behavioural therapy
		Dialectical behaviour therapy
		Family therapy/couples therapy/community reinforcement
		Therapeutic communities/residential rehabilitation
		Social behaviour and network therapy
		Psychodynamic therapy
		Relapse prevention
		Mentalisation‐based therapy
	12‐step facilitation therapy	
Behavioural incentives	Contingency management	Community reinforcement approach
	Cue exposure treatment	
	Non‐contingent rewards	
Self efficacy		12 step programmes
Self help/mutual aid		SMART

#### 2.2.2 Psychosocial Interventions and Adults Experiencing Homelessness

Psychosocial interventions are often used to address problematic substance use, poor mental health, and offending behaviours, as well as wider social determinants of health such as housing instability and homelessness, worklessness, and poor skills or education. As adults experiencing homelessness may be dealing with more than one of these issues at any given time, many will access services that include psychosocial interventions. It is therefore essential to understand the experiences of adults experiencing homelessness when accessing these interventions, and what might facilitate improved access. It is also important to understand which factors are perceived to be related to intervention effectiveness by adults experiencing homelessness.

### 2.3 How the Intervention Might Work

Broadly speaking, the main mechanism of change underpinning these interventions is psychological, focusing on the individual's psychological development and interaction with their social environment. However, this is a very broad mechanism, and does not help policy makers or practitioners to understand how to better design, implement, and deliver such interventions. There is no single theory of change underpinning these types of interventions; some are more explicitly based on formal theories, others less so (England et al. 2015), and others argue that psychosocial interventions draw on different theoretical models. In some areas, there are many different interventions derived from the same theoretical model.

In the protocol for this study (O'Leary et al. 2022), we drew on the work of Scott and colleagues (Scott, under review) to outline three meta‐theories that may underpin all psychosocial interventions. As part of this review, we extracted data from each included study to assess whether the study interventions drew on explicit theories of change, and if so, whether and how these theories related to the three meta‐theories outlined in the protocol. We report on this analysis later in this review.

### 2.4 Why It Is Important to Do This Review

#### 2.4.1 Policy Relevance

As previously noted, homelessness is a significant and growing policy issue in a number of high‐income countries around the world. It is increasingly recognised that homelessness has a devastating effect on those experiencing it, on the wider community, and on the public purse. There is an ongoing debate as to which interventions are most effective at preventing and reducing homelessness and the harms associated with homelessness.

Psychosocial interventions increasingly play a role in policy and practice responses to homelessness and the harms caused by homelessness. There is some evidence about the effectiveness of interventions in the general population, but not specifically in relation to adults experiencing homelessness. There is a limited but growing evidence base about the factors affecting access to and use of psychosocial interventions generally, but there is no evidence specifically related to adults experiencing homelessness.

There is also a significant gap in the current evidence base in terms of the voices of people with lived experience of homelessness, as it largely treats them as passive research participants. This review aims to elevate the voices of people with lived experience in three ways. First, there was an ‘experts by experience’ review process that ran alongside the technical peer review process. This enabled the review team to gain views on the relevance and appropriateness of the review and its outcomes to the users of services. Second, the team worked with a panel of people with lived experience to co‐produce the discussion, recommendations, and conclusions of the published review. Third, this review focuses specifically on the experiences of people experiencing homelessness as they access and use psychosocial interventions and thus aims to hear directly the voices of people with lived experiences, as collected in the included studies. We used the Guidance for Reporting Involvement of Patients and the Public (GRIPP2) (Staniszewska et al. 2017) process to report how we engaged with people experiencing homelessness in the design, conduct, reporting, and developing policy and practice recommendations arising from this review. The report is set out in the Appendix to this review.

#### 2.4.2 Previous Reviews

There are no systematic reviews that focus on the experiences of accessing or using psychosocial interventions for people experiencing homelessness. However, there are three published reviews that explore the experiences of people experiencing homelessness that are in part relevant to our review. Carver et al. (2020) provide a meta‐synthesis, centred on client engagement with youth homelessness, explored a range of contextual factors that defined the success or failure of the implementation of an intervention. Curry et al. (2021) provided a systematic review on what constitutes problematic substance misuse, combined with a meta‐ethnography (as utilised by one of the included studies in this review), which was useful as it also conducted a qualitative synthesis of the participants' views. Finally, Magwood et al. (2020), reviewed and evaluated harm reduction interventions with a focus on homeless populations, and had a similar focus on outcome measures such as social well‐being and potential treatment barriers. Each is driven by different research objectives, examines different interventions, and utilises different synthesis approaches, and therefore serves as a complement to this review.

## 3 Objectives

This systematic review is part of a broader evidence synthesis which intends to produce two reviews to address a significant gap in the evidence base identified by Luchenski et al. (2018) and by White and Narayanan (2021). The review published here is of qualitative data and used thematic synthesis to analyse the experiences of adults experiencing homelessness when accessing and using psychosocial interventions.

This review aimed to answer the following research questions:

1. What are the experiences of study participants when accessing or using psychosocial interventions?
2. Whether and how adults experiencing homelessness perceive the interventions work for them?
3. To what extent do these experiences vary by type of intervention, context, setting, geographical location, whether they are individual or group based, or whether they are stand alone or integrated with other interventions?
4. What are the explicit theories of change underpinning psychosocial interventions?

## 4 Methods

This review largely draws on the Homelessness Implementation Evidence and Gap Map (EGM) third edition (Singh and White 2022). The protocol for our review was published in the summer of 2022 (O'Leary et al. 2022).

### 4.1 Criteria for Considering Studies for This Review

#### 4.1.1 Types of Studies

The SPIDER framework (Cooke et al. 2012) was used in the development of the criteria for considering studies for this review. The following paragraphs set out the Sample, Phenomenon of Interest, Design, Evaluation, Research type.

##### 4.1.1.1 Sample

There are a number of definitions of homelessness available, reflecting differences between countries and over time. There are also different forms of homelessness, taking into account the length of time someone has been experiencing homelessness, distinctions between living on the street or in their vehicles, or having a temporary place to stay.

During the scoping work to develop the protocol for this study (O'Leary et al. 2022), we facilitated a workshop with five individuals with lived experience of homelessness to consider definitions, criteria for inclusion and exclusion, and the process of conducting this review. Following recommendations from those involved in this workshop, we used an adapted and widened definition of homelessness developed by Keenan et al. (2021). The revised definition for this review is:

Homelessness is defined as those individuals who are in inadequate accommodation (environments which are unhygienic and/or overcrowded), who are sleeping rough (sometimes defined as street homeless or unsheltered), those in temporary accommodation (such as shelters and hostels), those in insecure accommodation (such as those facing eviction or in abusive or unsafe environments), and people whose accommodation is inappropriate (such as those living in tents or vehicles, or ‘sofa surfing’).

The definition and inclusion criteria for homelessness shared great parity with that of the EGM, that is, including both the literal forms of homelessness and those at risk of homelessness, as such, it is unlikely to have resulted in significant divergences in search strategies/results. Our focus was on adults (men and women aged 18 years and over), undertaken in any high‐income country and published in English. Studies of families or children were excluded from the review, as in many countries (particularly the United Kingdom), there are different legal frameworks that apply to families and children experiencing homelessness, and thereby their access to different types of services, and different outcomes expected.

##### 4.1.1.2 Phenomenon of Interest

The review focused on formal psychosocial interventions used with adults experiencing homelessness. Interventions based solely or mainly on pharmacological approaches or approaches other than psychosocial were excluded. In developing the protocol for this review (O'Leary et al. 2022), we set out our definition of psychosocial interventions and a typology of interventions that we considered to meet this definition. This typology is presented again in the Appendix.

##### 4.1.1.3 Design

Eligible studies were those that used individual and group interviews, focus groups, observations, or other qualitative‐related methods focused on the experiences, views, or opinions of adults experiencing homelessness.

##### 4.1.1.4 Evaluation

We included studies where empirical data presenting the experiences, views, perspectives or opinions of people who are experiencing homelessness or at risk of homelessness when accessing or using psychosocial interventions, and are directly presented either as direct quotes or summaries, or as reports of participant experiences by researchers.

##### 4.1.1.5 Research Type

Eligible studies included data reported either as part of a mixed methods or qualitative study about the use of psychosocial interventions focused on people who are experiencing homelessness.

### 4.2 Search Methods for Identification of Studies

#### 4.2.1 Electronic Searches

Studies included in this review were identified in three ways:

- The Homelessness Implementation Studies Evidence and Gap Map (third edition) [38] (set out below);
- call for evidence (set out in the section ‘searching other sources’ below); and
- hand searches (set out in the section ‘searching other sources’ below).

##### 4.2.1.1 The Homelessness Implementation Studies EGM

The main source of studies included in this review was from the Implementation EGM third edition, published by The Campbell Collaboration (Singh and White 2022). The searches for this updated EGM were conducted by a team from the Campbell Collaboration between March and September 2021. The EGM included 401 primary studies and systematic reviews. The process for undertaking these searches (including search strings) is set out in the published protocol for the Implementation EGM (Singh et al. 2023). This states that the review included a broad range of qualitative studies, mixed method studies, and quantitative studies that reported on implementation issues. The team reported that they searched several databases, websites and registries, conducted backward citation tracking of included studies, contacted researchers and hand‐searched a number of journals. The team also reported that, because many relevant evaluations are unlikely to be published in academic journals, an extensive grey literature search was undertaken. The protocol includes details of the websites searched for grey literature.

Two reviewers independently undertook a title and abstract search of each of the studies listed in the EGM. Each title and abstract was reviewed against the inclusion and exclusion criteria for this review. The two reviewers then compared their assessments. One systematic review was identified through this process, and this was unpacked to identify additional primary studies. These were also subject to title and abstract reviews by two reviewers independently. Studies that appeared to meet the inclusion criteria, or where there was disagreement between the reviewers, were subjected to a full‐text review, which was again undertaken independently by two reviewers. Any disagreements at this stage were referred to a third reviewer for decision.

#### 4.2.2 Searching Other Resources

In January/February 2022, a call for grey evidence was circulated by our review team. The call was disseminated through Manchester Metropolitan University and the Centre for Homelessness Impact, inviting people with lived experience, researchers, commissioners, service providers and wider stakeholders to submit relevant grey literature evidence for consideration in both these parallel reviews.

Specifically, the call was for evidence that is:

- Empirical, based on research that:
  ∘ elevates the voice of people with experience of homelessness;
  ∘ measures the impact of interventions (before and after, quasi‐experimental, randomised controlled trial) [1]; or
  ∘ identifies the experiences of people with experience of homelessness when they access and use psychosocial interventions [2];
- about psychosocial interventions aimed at preventing or reducing homelessness, mental ill‐health, and problematic substance use;
- not published in a book or academic journal; and
- specific to the United Kingdom, or England, Northern Ireland, Scotland or Wales.

The Call for Evidence did not result in any relevant studies being identified.

The reviewers also undertook a hand search of key, subject‐specific journals, using similar search terms as the EGMs. Hand searches are considered an important part of a systematic review, as they reduce the risk of poorly or inaccurately indexed articles and to ensure that no substantive relevant evaluation or study is missed by the search strategy. It should be noted that given the extensive searches undertaken by The Campbell Collaboration for the three published EGMs, the hand searches undertaken by our review team focused on a small number of the most important journals in the field. The hand‐searched journals included:

- *Psychiatric Services Journal*
- *American Journal of Public Health*
- *BMJ*
- *European Journal of Homelessness*
- *Housing Studies*
- *Social Policy and Administration*
- *Journal of Social Distress and Homelessness*

Hand searches were conducted independently by two reviewers, using the inclusion and exclusion criteria developed for this review. The reviewers compared the results of their hand searches to reach agreement on which studies appeared to be relevant. Following removal of studies already included in the EGM, additional studies identified through the hand searches were subjected to title and abstract review as set out above.

1. And therefore relevant only to the effectiveness review running alongside the review published here.
2. And therefore relevant to this review.

### 4.3 Data Collection and Analysis

#### 4.3.1 Data Extraction and Management

First, descriptive data were extracted from included studies by two reviewers. These data included the details of the study, description of the theory of change underpinning the intervention, description of the intervention, qualitative data collection method used, qualitative analysis method used, and the following specific data:

- Publication details (e.g., authors, year, source);
- Geographical location (country);
- Setting;
- Intervention details, including basis, focus, typology classification, explicit theory of change;
- Participant details, including classification (e.g., age, gender, ethnicity, disability, whether service user);
- Number of service users involved in the study;
- Research aim and design.

Second, two reviewers independently extracted the findings from the included studies. Each reviewer examined the findings section of each of the included studies, extracting whole sentences that were or contained direct quotes from participants with lived experience of homelessness, or that were or contained authors' interpretation of the experiences, perspectives, or views.

In relation to the extraction of direct quotes from participants, three of the included studies only involved participants who were accessing and using the intervention and had experience of homelessness. In all three cases, all quoted material was extracted. Four of the included studies also included participants who were not individuals experiencing homelessness. In each case, the study authors distinguished between participants who were clients or service users and other participants (e.g., staff, stakeholders, programme staff), which facilitated data extraction.

The extraction of author interpretation data was less straightforward. Often, authors would make clear that they were presenting the views of individuals or groups of individuals with lived experience. Authors would reference service users, participants, or clients, for example, discussing their views and experiences. Other times, it was less than clear.

In both cases, data extracted by each reviewer were compared, and any differences were identified. These were discussed by the two reviewers to achieve consensus on whether data should be included in the review. Once consensus was achieved, extracted data was uploaded to NVivo version 12 for line‐by‐line coding (first stage of analysis) for thematic synthesis analysis of participant experience data, and subsequently used for stages 2 and 3 of the thematic synthesis analysis.

##### 4.3.1.1 Appraisal of the Methodological Limitations of the Included Studies

Eligible studies that are included in the Implementation EGM have already been assessed using Campbell's Critical Appraisal Tool for Primary Studies (White and Narayanan 2021, 60). The appraisal considers the following questions:

1. Are the research questions clearly stated?
2a. Is the qualitative methodology adequately described?
2b. Is the qualitative methodology appropriate?
3a. Is the recruitment/sampling strategy adequately described?
3b. Is the recruitment/sampling strategy appropriate?
4. Has the relationship between researchers and participants been adequately considered?
5. Have ethical considerations been sufficiently considered?
6. Is the data collection approach adequately described?
7a. Is the data analysis approach adequately described?
7b. Is the data analysis sufficiently rigorous?
8. Is there a clear statement of policy recommendations or implications of the research?
9. Are the findings or recommendations based on the report findings?

**Figure 1:**
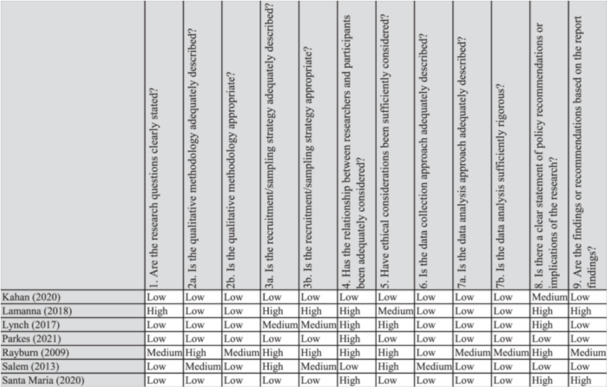
Individual study‐level quality assessment.

This assessment was undertaken by the Campbell team in their development of the EGM. We did not undertake any further assessment of the methodological limitations of those included studies that were identified from the EGM. For any additional studies identified through either the hand searches of relevant journals, the call for grey literature, or through unpacking of systematic reviews, we conducted an assessment of confidence in the findings using this same tool. For these additional studies, classifications were undertaken by one researcher and judgements (high/medium/low confidence) were verified by a second researcher. These quality assessments were not used as a criteria in our decisions around inclusion of studies in this review. The individual study‐level quality assessments are reported in Figure 1.

##### 4.3.1.2 Analysis

There are a number of different methods available for undertaking qualitative evidence syntheses. The review team used thematic synthesis (Thomas and Harden 2008) for a number of reasons. First, this method was deemed appropriate because of the focus of the review on the experiences (Carroll 2017) of people experiencing homelessness as they access and use psychological interventions, although the review team recognises that a number of methods would be appropriate for this objective. More importantly, thematic synthesis seemed the most appropriate approach because of its perceived neutrality in terms of the epistemology of underlying studies (Booth 2017), and because it is appropriate given that the review is aimed at policy makers, practitioners, and those involved in designing interventions (Barnett‐Page and Thomas 2009). Finally, for practical reasons, because of time and available resources and the expertise of the review team, thematic synthesis was more appropriate than other methods such as meta‐ethnography (Booth 2017).

Thematic synthesis (Thomas and Harden 2008) involves three analytic and inductive steps. The first step involves line‐by‐line descriptive coding of the data from the findings of the primary studies. This involves examining each sentence individually, and giving it one or more short descriptive codes (McMahon et al. 2022). This coding was undertaken by two reviewers, independently. Each reviewer distinguished between direct quotes from study participants with lived experience of homelessness and study authors' interpretation of participants' views, perceptions, and experiences. Once each study had been coded line by line, the two reviewers met to compare descriptive codes and coded material, discuss any differences, and agree on an overall coding framework (presented in the Appendix).

The second stage of analysis involved identifying possible relationships between the descriptive codes, and thus organising the descriptive codes into a series of descriptive themes. These themes describe the experiences of participants as they engage in psychosocial interventions. The reviewers independently developed descriptive themes; the lead reviewer developed 14 and the second reviewer developed 11 descriptive themes. The reviewers met several times to discuss the relationships that were identified and the development of the descriptive themes, using mind maps and other visual methods, reviewing these against the material coded under each of the 1st stage descriptive codes, until agreement was reached. (There was good agreement between the reviewers in most cases; we have highlighted in the analysis section where major differences arose between the reviewers and where reaching agreement was a more iterative process.) Each of the resulting descriptive themes was then presented, with illustrative extracts from the coded material, at a workshop with a panel of individuals with lived experience of homelessness, and changes were made when appropriate. Through this iterative and inclusive process, a final agreement was reached on the descriptive themes presented in this review.

The third step in thematic synthesis is ‘the most difficult to describe and, potentially, the most controversial’ (Thomas and Harden 2008), and is the development of analytical themes. Whereas the first two stages outlined above are intended to describe and summarise data extracted from the included studies, this third stage of analysis ‘goes beyond’ (Thomas and Harden 2008) the primary studies to directly address the questions set out for the review. However, stages 2 and 3 also overlap with each other, as often review questions can be answered during stage 2 analysis (McMahon et al. 2022; Thomas et al. 2017). It involved iteratively examining the descriptive themes, drawing out and inferring from these themes the experiences of participants, their perceptions of whether and how the intervention worked for them, and whether their experiences varied by type of intervention, location, and context. The analytical themes were also discussed and evaluated in a workshop held with a panel of people with first‐hand experience of homelessness, held in early 2023.

##### 4.3.1.3 Experiences

The core focus of this review was to understand the experiences of, views about, perspectives on, and opinions of adults experiencing homelessness when they (a) access and (b) use psychosocial interventions, and what factors they perceive are important to the effectiveness of psychosocial interventions. The evidence set out in the background section of this review clearly demonstrates that adults experiencing homelessness have significantly higher levels of mental ill‐health, problematic substance use, and housing instability compared to the general population, and are also less likely to be able to access services (particularly general healthcare) to reduce the impact of, and negative effects from, these experiences. By focusing this review on the experiences of adults experiencing homelessness, the review aims to elevate the voices of people experiencing homelessness and thereby address a fundamental limitation of many systematic reviews – that of treating service users as passive research participants rather than as citizens who may actively participate in their own recovery.

##### 4.3.1.4 Reflexivity Statement

The two researchers involved in screening and eligibility decisions both have a background in homelessness research, including evidence synthesis and primary research that has involved working with people with first‐hand experience of homelessness. Both researchers have experience working in front‐line services aimed at, and accessed by, individuals with first‐hand experience of homelessness, including individuals experiencing homelessness at the time.

One of the two researchers involved in screening and eligibility decisions was also involved in data extraction and analysis. The other researcher has a background in social work, both academic and practice‐based, and considerable experience in evidence synthesis.

None of the researchers involved have had first‐hand experience of homelessness. All researchers are committed to involving individuals with first‐hand experience in the design of research and using research to elevate the voice of individuals with first‐hand experience of homelessness.

## 5 Results

### 5.1 Description of Studies

#### 5.1.1 Results of the Search

The searches identified 468 primary studies, of which 7 were included in this review. Qualitative data from these included studies provided data from 84 adults with experience of homelessness who were accessing or using psychosocial interventions, and three who were accessing treatment as usual services. Figure 1 sets out the results from the searches, title and abstract reviews, full text reviews, and decisions on the inclusion of studies.

#### 5.1.2 Included Studies

We identified seven studies that met our inclusion criteria. The characteristics of these studies are set out in Table 2.

**Table 2:** Characteristics of included studies.

Author	Country	Setting	Sample characteristics	Number of participants	Intervention	Method	Theory of change	Research aims
Kahan et al. (2020)	Canada	Covenant House, Toronto	Female‐identified survivors of domestic violence experiencing homelessness, aged 19–24	12	Peer Education and Connection through Empowerment (PEACE), a time‐limited, trauma‐informed, peer‐supported psychosocial group intervention	Interviews Thematic analysis Qualitative	Reference to previous research	Examine the acceptability of the intervention
Lamanna et al. (2018)	Canada	Individuals who have been discharged for one of three hospitals in Toronto	Adults experiencing homelessness, average age 42 (±9.8)	22	Co‐ordinated Access to Care for Homeless People (CATCH), brief intervention aimed at improved continuity of care	Focus groups and interviews Thematic analysis Part of mixed methods	Reference to previous research	Explore perspectives on the role of the intervention
Lynch et al. (2017)	Canada	Three community shelters in Calgary and Toronto	Young people experiencing homelessness, average age 19.3 years	6 (3 treatment, 3 control)	Motivational interviewing	Interviews Thematic analysis Qualitative	Explicit theory of change	Determine the effectiveness of a resilience and motivational intervention to increase engagement with street‐involved youth
Parkes et al. (2021)	Scotland	Wellbeing Centre, Edinburgh	Adults experiencing homelessness	10	Psychosocial group‐work programme	Interviews and documentary analysis Framework analysis Qualitative	No	Document service changes because of Covid
Rayburn and Wright (2009)	USA	First steps programme, Orlando, Florida	Men experiencing homelessness	10	12 Step programme	Interviews Qualitative	Reference to previous research	Tell the story of recovering homeless individuals in a 12‐Step programme
Salem et al. (2013)	USA	Residential rehab centre in Southern California	Women ex offenders	14	Residential rehabilitation	Focus groups Content analysis Qualitative	No	Understand experiences of homeless female ex‐offenders post‐prison release
Santa Maria et al. (2020)	USA	Homeless shelter in Houston, Texas	Young people experiencing homelessness, aged 18–21	10	Mindfulness‐Based Intervention	Baseline online survey and interviews. Mixed methods study	Explicit theory of change	To build on the existing feasibility literature assess the feasibility and acceptability of a slightly modified intervention

##### 5.1.2.1 Participant Characteristics

The included studies cover 84 participants with experience of homelessness, of which 81 were accessing or using psychosocial interventions, and 3 in 1 study (Lynch et al. 2017) were accessing a comparator intervention. (For six of the included studies, the number of participants was clearly and explicitly stated. Identifying for number of participants in one study – Santa Maria et al. (2020) – was less straightforward. This was a mixed methods study and stated that the number of participants was *n* = 39. However, the data extracted from this study related to the qualitative findings only, and we determined that this part of the study involved 10 participants.)

Two studies involved 26 women participants (Kahan et al. 2020; Salem et al. 2013), of whom 12 were female‐identified survivors of domestic violence aged under 25 (Kahan et al. 2020) and 14 were women who had been in prison (Salem et al. 2013). One study focused on men experiencing homelessness, covering 10 participants (Rayburn and Wright 2009). Two studies focused on adults (both men and women). Lamanna et al. (2018) included 22 participants experiencing homelessness as they were discharged from hospital, and Parkes et al. (2021) 10 participants with substance use issues. Table 3 provides a breakdown of the number of participants by sex for each study.

**Table 3:** Breakdown of participants by study and sex.

Study	Women participants	Men participants	Total participants
Kahan et al. (2020)	12		12
Lamanna et al. (2018)	6	16	22
Lynch et al. (2017)	3	3	6
Parkes et al. (2021)	2	8	10
Rayburn and Wright (2009)		10	10
Salem et al. (2013)	14		14
Santa Maria et al. (2020)			10

*Note:* Lynch et al. (2017) reports 3 participants receiving the intervention and 3 accessing a comparator intervention. Santa Maria et al. (2020) reports 39 participants included in a mixed methods study. Ten participants completed an exit interview, which is the basis of the qualitative findings reported. The study authors do not provide a breakdown of the sex of the 10 participants.

Two studies focused on young people experiencing homelessness. Kahan et al. (2020) on 12 young women aged 16–24 escaping domestic violence, and Santa Maria et al. (2020) on 10 participants aged 18–21.

Two of the included studies provided details of the participants' race. Rayburn and Wright (2009) state that of the 10 participants, 8 were Black, 1 Hispanic and 1 White. Salem et al. (2013) state that of the 14 participants, African American (79%), followed by White (14%) and Hispanic/Latino (7%). The other five include studies do not provide information on the race of participants.

##### 5.1.2.2 Geographical Location of Studies

Three studies took place in Canada (Kahan et al. 2020; Lamanna et al. 2018; Lynch et al. 2017), all in Toronto (Lynch et al. (2017) included shelters in Toronto and Calgary). Three studies took place in the United States: in Florida (Rayburn and Wright 2009); California (Salem et al. 2013); and Texas (Santa Maria et al. 2020). One study took place in Edinburgh, Scotland (Parkes et al. 2021).

##### 5.1.2.3 Intervention Characteristics

Interventions covered included brief intervention (Lamanna et al. 2018), motivational interviewing (Lynch et al. 2017), 12‐step programme (Rayburn and Wright 2009), and residential rehabilitation (Salem et al. 2013). Kahan et al. (2020) and Parkes et al. (2021) both described the interventions as group psychosocial interventions, and Santa Maria et al. (2020) as a mindfulness‐based intervention.

##### 5.1.2.4 Study Designs

Five of the studies were qualitative (Kahan et al. 2020; Lynch et al. 2017; Parkes et al. 2021; Rayburn and Wright 2009; Salem et al. 2013). One study was described as being a subset of a larger mixed methods study (Lamanna et al. 2018); and one study was described as a mixed methods feasibility study (Santa Maria et al. 2020).

All but two studies (Rayburn and Wright 2009; Santa Maria et al. 2020) stated the analytical method used to generate findings. Thematic analysis was used in three Canadian studies (Kahan et al. 2020; Lamanna et al. 2018; Lynch et al. 2017). Parkes et al. (2021) stated that framework analysis was used, and content analysis was used by Salem et al. (2013).

Six studies used interviews as the data collection method, of which three (Kahan et al. 2020; Lynch et al. 2017; Rayburn and Wright 2009) used this as the only method and three used it in conjunction with other approaches. Lamanna et al. (2018) used interviews and focus groups, Parkes et al. (2021) used interviews and analysis of policy/service documents, and Santa Maria et al. (2020) utilised interviews and baseline surveys. One study (Salem et al. 2013) used focus groups as the only method for collecting data.

#### 5.1.3 Excluded Studies

Ten papers were excluded following full text review. Details of these studies and the reasons for exclusion are set out in Table 4.

**Table 4:** Characteristics of excluded studies.

Authors	Title	Reason for exclusion
Ayer et al. (2018)	Evaluation of the Connections to Care (C2C) Initiative	Does not include any experiences of individuals with first hand experience of homelessness
Cormack (2009)	Counselling marginalised young people: a qualitative analysis of young homeless people's views of counselling	Not a relevant intervention
Goradietsky (2020)	Development of a strengths and empowerment‐focused intervention for marginalised populations with a focus on women, low‐income, and racial‐ethnic minorities	Not a relevant intervention
Leonard et al. (2017)	‘Coming From the Place of Walking with the Youth‐that Feeds Everything’: a Mixed Methods Case Study of a Runaway and Homeless Youth Organisation	Not a relevant intervention
McCay et al. (2016)	Toward treatment integrity: developing an approach to measure the treatment integrity of a Dialectical Behaviour Therapy intervention with homeless youth in the community	Does not include any experiences of individuals with first hand experience of homelessness
McGraw et al. (2010)	Adopting best practices: lessons learned in the Collaborative Initiative to end Chronic Homelessness (CICH)	Does not include any experiences of individuals with first hand experience of homelessness
MissionAustralia (2016)	Room to grow final evaluation report	Not a relevant sample/population
Rayburn and Wright (2010)	Sobering up on the streets: homelessness men in Alcoholics Anonymous	Same participant sample as already included study
Schwaiger and Williamson (2021)	The art of mentalising: a mentalisation‐based art initiative with homeless people within a psychological informed environment	Does not include any experiences of individuals with first hand experience of homelessness
Yakovchenko et al. (2021)	Implementing a complex psychosocial intervention for unstably housed veterans: a realist‐informed evaluation case study	Does not include any experiences of individuals with first hand experience of homelessness

##### 5.1.3.1 Use of Theory in the Included Studies

The fourth research aim was to describe what explicit theories of change underpinned the psychosocial interventions being evaluated in the included studies. This objective was an important part of the review process because: (1) of the lack of any single agreed definition of what a psychosocial intervention is; (2) the lack of a single explanation of how these interventions work; and (3) policy makers and practitioners need to understand how these interventions work when they design, implement, and deliver psychosocial interventions.

For each of the seven included studies, we examined whether and how the authors explained how the intervention might work. Each study was coded by the lead reviewer. This coding covered: whether an explanation was provided for how the intervention would work; whether a justification was provided for the intervention; and whether and how this related to the three meta‐theories identified in the ‘how the intervention might work’ section of this report. Three relevant theories were identified in the protocol for this review (O'Leary et al. 2022) and were:

1. Interpersonal Relationships – relationships as a driver of homelessness;
2. Habituation – behaviour disruption;
3. Meta Cognitive Awareness – cognitive approaches, that is, dysfunctional beliefs.

None of the included studies reference any of the three meta‐theories identified in the protocol for this study.

Of the seven included studies, two include an explicit theory of change detailing the intervention design and how it is intended to work. Lynch et al. (2017) evaluate a multi‐component intervention aimed at young people experiencing street homelessness, which combined resilience‐based approaches and motivational interviewing. The authors describe the intervention and provide details of the two theoretical models underpinning its design, the Seven Cs Model of Resilience (Ginsburg and Jablow 2006, as cited in Lynch et al. 2017) and motivational interviewing (Miller and Rollnick 2002, as cited in Lynch et al. 2017). The authors state that: ‘The intervention was designed to enhance the capacity of street‐involved youth by encouraging their engagement in health‐promoting relationships, reducing risk‐oriented behaviors, and promoting the acquisition of skills and healthy behaviors to support transition to young adulthood’. Santa Maria et al. (2020) provide a more detailed insight into the theory of change underpinning the intervention, a mindfulness‐based intervention for young people experiencing homelessness. The authors reference two theoretical approaches, risk amplification model (Meyer 2003, as cited in Santa Maria et al. 2020) and minority stress model (Whitbeck et al. 1999, as cited in Santa Maria et al. 2020). The authors provided detailed justification for the use of these models, and illustrate the key components of the intervention and the intended outcomes on page 264.

Three of the included studies reference previous research to justify the design and intent of the evaluated intervention. Kahan et al. (2020) provide details on the design of the intervention, locating this design in trauma‐informed practices, and providing brief details on why these principles are used. Lamanna et al. (2018) provide a detailed explanation of the level of need within their population of interest (in this case, adults experiencing homelessness as they are discharged from hospital), and describe the structure of the evaluated intervention. The study references previous research by several of the study authors on brief interventions. Rayburn and Wright (2009) provide a detailed literature review around 12‐step programmes (in this case, specifically Alcoholics Anonymous) in justification of the design of the evaluated intervention. The authors also provide details of the specific structure and components of the First Steps programme that is the focus of their research.

Two of the included studies did not provide any justification for the design or intent of the evaluated intervention. In a study of service change because of Covid‐19 at a homelessness centre in Edinburgh, Parkes et al. (2021) provide details of the services provided, and states that the centre adopts a harm reduction approach and includes a ‘psychosocial’ programme, though do not reference the extant literature or provide any details to justify these approaches. The authors also state that their research was informed by two theoretical approaches, namely psychologically informed environments (PIEs) and enabling environments. Salem et al. (2013), in an evaluation of a residential rehabilitation programme targeting women experiencing homelessness who had been in prison, provide details of the model underpinning the design and conduct of the research. The authors do not provide any insight to justify the intervention design.

### 5.2 Synthesis of Results

**Figure 2:**
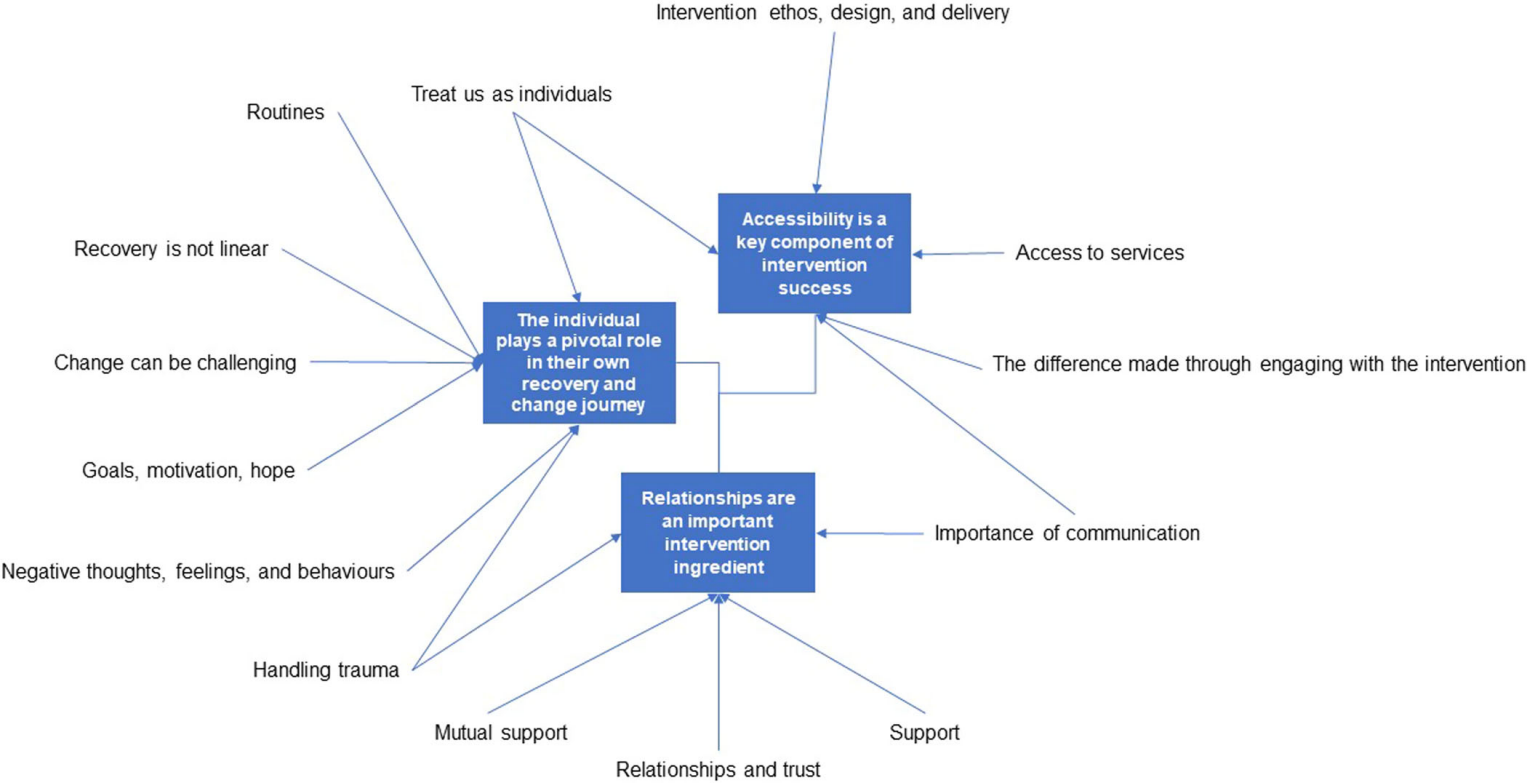
Descriptive and analytical themes.

Three analytical themes were developed from the 14 descriptive themes through an iterative and reflective process. Three of the descriptive themes contributed to more than one analytical theme, although generally descriptive themes contributed to a single analytical theme. These themes are: (1) the individual plays a pivotal role in their recovery and change journey; (2) accessibility is a key component of intervention success; (3) relationships are an important intervention ingredient. Figure 2 illustrates the connectivity between the 14 descriptive themes and these 3 analytical themes.

Here, we set out each of the three analytical themes and present the findings from our analysis that support each of these themes. Of the fourteen descriptive themes, six directly answered one of the research questions underpinning this review. These six are reported here as findings under the relevant analytical theme to which they contribute.

#### 5.2.1 Theme 1: The Individual Plays a Pivotal Role in Their Own Recovery and Change Journey

The aim of psychosocial interventions covered by this review is to promote change in behaviour or cognition for individuals with lived experience of homelessness, and that these changes lead to reduced substance use, improved housing stability, or improved mental health. The individuals accessing and using these psychosocial interventions, therefore, play a pivotal role in engendering this change. Their sense of hope, their motivation to achieve change, and the goals they set themselves are as important to success as the design and delivery of the intervention itself. These individuals have thoughts, feelings and behaviours that are fundamentally important to the sense of hope and their motivation. These thoughts, feelings, and behaviours can provide powerful motivations for change, but can also create barriers or challenges.

The pivotal role of the individual in this change journey is reflected through a fundamental need to be treated as individuals, for services and interventions to take account of each individual's unique experience of homelessness, their challenges, goals, hopes and dreams. But it goes further than this, with a key ingredient of successful psychosocial interventions being the extent to which service users are actively involved in decisions about the design of these services.

##### 5.2.1.1 Finding 1: An Individual's Goals, Motivation, and Sense of Hope Are Important to Enabling Change

This finding is developed in all seven of the included studies, and talks directly to the review's second research question about what makes for effective interventions. The finding recognises that experiencing homelessness can have a negative impact on an individual's sense of self worth and sense of hope, and that motivation is an important ingredient in an individual's change journey.

##### 5.2.1.2 Finding 2: An Individual's Negative Thoughts, Feelings, and Behaviours Can Adversely Impact Their Change Journey

This finding speaks to the role that negative feelings and thoughts can play, of powerlessness and being out of control, and how this can adversely affect an individual's change journey. It is about the role that the intervention – and particularly relationships with peers and staff during the intervention – can play in challenging negative thoughts and feelings, and augmenting self‐awareness. This finding was developed from six studies (all but Salem et al. 2013), although none of the study‐level descriptive themes relates to negative feelings, thoughts, and behaviours.

##### 5.2.1.3 Finding 3: Recovery Is Not Linear

This finding speaks to recovery or the process of change as a journey, with many bumps and different turns along the route. In particular, an individual's change journey is not a straight line of progression from homelessness to being stably housed, and it is important the both the intervention design and how it is delivered recognises this. During the workshop with a panel of individuals with first‐hand experience of homelessness to discuss the descriptive and analytical themes, one panel member used the analogy of ‘a game of snakes and ladders’ to summarise this descriptive theme. In this game, an individual's progress from start to finish often involves episodes of rapid positive progress (ladders) and also the reverse when the individual lands on a snake. In both cases, this is outside the control of the individual player. This finding develops from four of the included studies (Lamanna et al. 2018; Lynch et al. 2017; Parkes et al. 2021; Salem et al. 2013) and addresses the first of the review's research questions.

#### 5.2.2 Theme 2: Accessibility Is a Key Component of Intervention Success

Accessibility relates to the geographical location and physical layout of services. It also relates to the ethos of the service or intervention; the extent to which participants felt welcomed, able to be open and honest, and did not feel judged. However, this analytical theme also recognises that many participants have wider experience of using both other homelessness services and mainstream services, and often face significant barriers and challenges when they do so. This wider experience may affect their initial engagement with psychosocial interventions.

It is also clear that the ethos of the intervention plays a fundamental role for many participants. Participants across a number of the included studies talked about how they valued the non‐judgemental space provided by the intervention: ‘There was also discussion about the physical space, with several participants talking about the location as being important, including its accessibility by public transport’.

##### 5.2.2.1 Finding 4: People Experiencing Homelessness Face Several Challenges When Accessing and Using Mainstream Services

Participants in four of the included studies (Lamanna et al. 2018; Lynch et al. 2017; Parkes et al. 2021; Salem et al. 2013) discuss the challenges they faced when trying to access mainstream services, and the consequences they experienced because of these challenges. These challenges arise both because of lack of signposting to, or knowledge of services and other interventions, but also because of the discrimination and stigma they experience when trying to access services. Although this finding does not directly relate to the first research question, which is specifically about access to psychosocial interventions, it nevertheless does speak to issues faced by people experiencing homelessness more generally about accessing services.

##### 5.2.2.2 Finding 5: A Non‐Judgemental Approach Is an Important Part of Accessibility

This finding addresses the second research question, as it speaks to what participants perceive to be important to making the intervention work for them. This was found in each of the seven included studies. Alongside the physical aspects, accessibility – such as its location, layout, and available facilities – participants identified that it was important to have a warm and inviting approach in which relationships between staff and service users were based on a non‐judgemental, genuine and open approach.

#### 5.2.3 Theme 3: Relationships Are an Important Intervention Ingredient

One aspect of the interventions that was consistently discussed across the included studies was that of the supportive relationship between intervention staff, particularly those with lived experience of homelessness, and service users. Key to this is the role that peer support can play as an active ingredient in successful psychosocial interventions. Several participants across a number of the included studies (Kahan et al. 2020; Lynch et al. 2017; Parkes et al. 2021) talk about the positive benefits to their change journey of peer support, with staff with lived experience providing inspiration, being accessible and approachable, and helping them to achieve goals.

Communication was seen to be a fundamental ingredient of this support relationship. Many of the participants across four of the studies discussed the frequency, type, and positivity of their communication with support staff, and that this made a positive contribution to their experience of, and engagement with, the intervention.

##### 5.2.3.1 Finding 6: Relationships and Trust Are Important Ingredients for Successful Psychosocial Interventions

The importance of relationships, with peers and support workers, and the role that trust plays in these relationships, is found in six of the seven included studies. (It is not found in Rayburn and Wright [2009].) Throughout the included studies, relationships and trust are seen as an important ingredient in how the intervention might work, and therefore addresses the second research question for this review. References to relationships and trust are also found in study‐level descriptive themes, identified by study authors through their analysis.

The review team was able to address two of the four research questions raised through this analysis, namely: (1) what are the experiences of study participants when accessing or using psychosocial interventions; and (2) whether and how adults experiencing homelessness perceive the interventions that work for them? The small number of included studies, the limited number of interventions covered by the included studies (and the lack of more than one study covering each intervention), and the fact that six of the seven studies were undertaken in North America, meant that we could not adequately address the third research question. The fourth research question is discussed in the section ‘use of theory in included studies’.

## 6 Discussion

### 6.1 Summary of Main Results

This systematic review aimed to understand the experiences of adults experiencing homelessness when they access and use psychosocial interventions. This group of interventions is increasingly of interest to policymakers and service providers, as there is growing evidence of their effectiveness among the general population. As such, it is essential to understand whether they work for adults experiencing homelessness. But given that this population often face significant challenges when accessing public services – bureaucratic barriers that prevent them from registering for, and accessing services, poor physical location of services that deter or prevent access, and stigma and discrimination – it is also vitally important to understand their experiences and how these experiences might affect whether and how they access and use psychosocial interventions, and whether these interventions work.

This review focused on qualitative and mixed methods studies that presented data on the experiences, views, perceptions, and opinions of adults experiencing homelessness as they accessed psychosocial interventions, and services more generally. The review found seven studies that met our inclusion criteria. The oldest of the studies dates to 2009; three studies were completed in Canada, three in the United States, and one in Scotland. Two studies explored the experiences of women experiencing homelessness, two the experiences of young people experiencing homelessness (of which one was about young women), and one of the studies focused on men. There were a number of different psychosocial interventions covered by the included studies. Two explored participants' views as they accessed group‐based psychosocial interventions. Two explored substance use interventions; one study looked at a 12‐step programme, and the second at a residential rehabilitation intervention. Brief interventions (one study), motivational interviewing (one study), and mindfulness‐based interventions (one study) were also explored in the included studies.

One of the objectives of this review was to understand any explicit theories of change underpinning the interventions or explaining the relationship between the inputs of the intervention and the outputs achieved for and with the participants.

Due to diversity in the included psychosocial interventions as noted above under ‘intervention characteristics’ and the lack of explicit theories of change described in the included studies, it has not been possible to shed further light on this, other than the extracted data relating to theories underpinning interventions in the section above on ‘use of theory in the included studies’.

**Figure 3:**
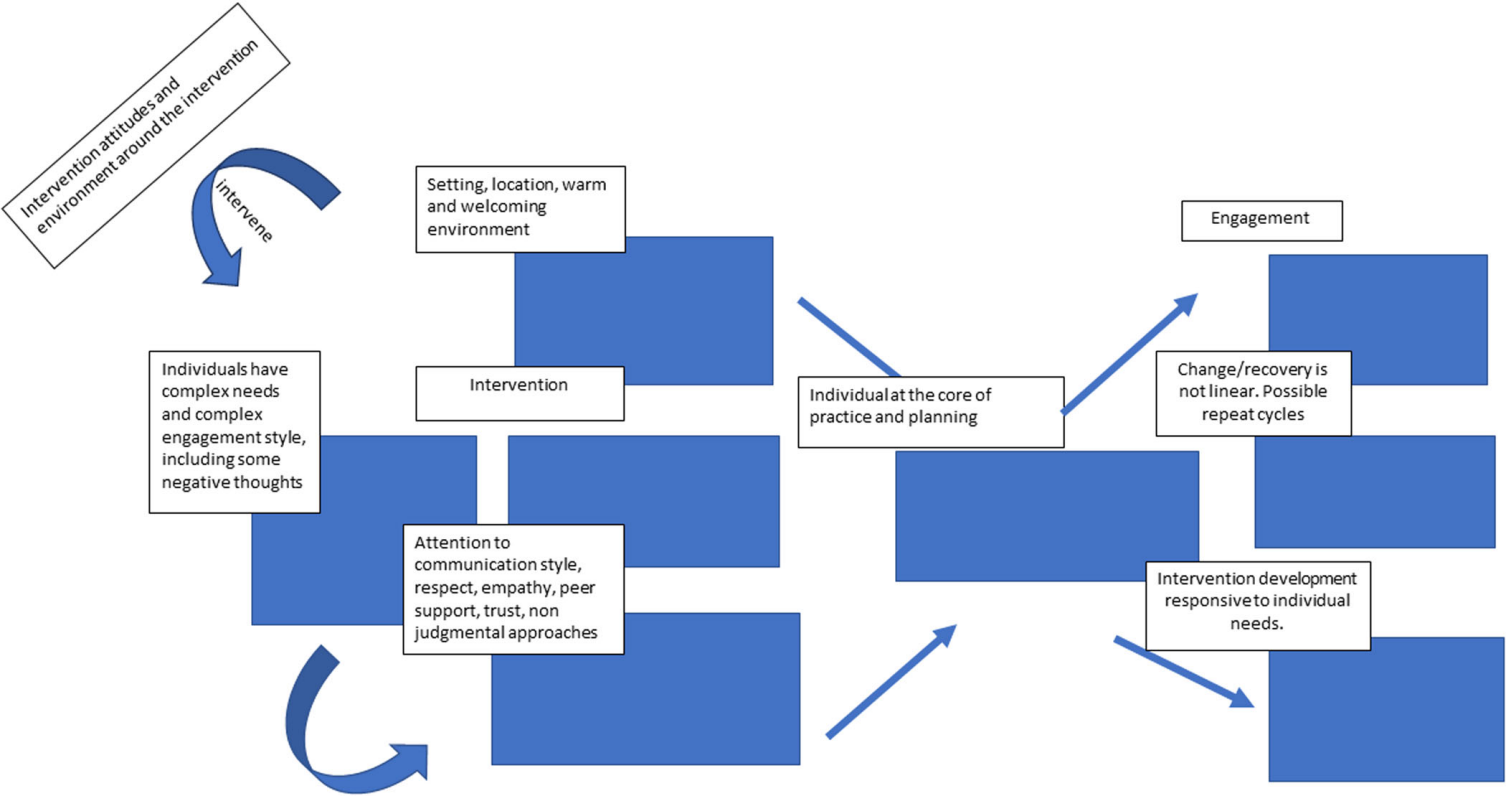
Emerging theory of change for psychosocial interventions.

However, based on data from the descriptive and analytical themes, from the perspectives of participants with lived experience, there are several key characteristics that promote their engagement with interventions. The logic model that follows is, therefore, a ‘theory of engagement’ that might contribute to planning future interventions to potentiate beneficial engagement as an intermediate outcome on a pathway to positive outcomes for people with lived experience of homelessness. This is illustrated in Figure 3.

### 6.2 Overall Completeness and Applicability of Evidence

The included studies only cover a small number of psychosocial interventions. For all but one type of psychosocial intervention, there is only one study for each type of intervention covered. Therefore, it was not possible to draw out any analysis or findings that are specific to any of the individual psychosocial interventions covered by the included studies. To some extent, this limitation is mitigated by the nature of the experiences data contained in the included studies; these data generally lack specificity to the individual psychosocial interventions being evaluated, and are focused on more general issues around relationships, experiences, access, and service provision.

This review is one of two being conducted by the review team around psychosocial interventions and adults experiencing homelessness. The second review, reported separately, examines effectiveness. The review team had hoped to undertake synthesis across these two reviews, to provide insight into both the effectiveness of specific interventions and the experiences of those accessing and using those interventions. While it has been possible to draw some general conclusions from synthesis about psychosocial interventions overall, intervention specific synthesis has not been possible. There are only two specific interventions for which there is both effectiveness and experiences evidence, namely motivational interviewing and brief interventions. In each case, there is a single included study in this review, and neither of these relates to a study included in the effectiveness review. This lack of complementary evidence significantly limits the policy lessons that can be drawn from the extant evidence.

Men are disproportionately represented in the effectiveness studies around psychosocial interventions, a finding that is consistent with other systematic reviews around the effectiveness of interventions for people experiencing homelessness (see, e.g., O'Leary et al., forthcoming). The experiences studies are, in comparison, much more evenly balanced, with 37 women and 37 men receiving the interventions of interest across the 7 studies (as set out in table x, details of the sex of 10 participants in one study were not provided). Two of the studies only involved women participants, and one only involved men. As women and men experience homelessness differently, and often experience homelessness for different reasons (for women, a significant driver of homelessness is experience of domestic violence), it is encouraging to see women's experiences of psychosocial interventions receiving this level of research attention.

Finally, six of the seven included studies are from North America, with one being from Scotland. This presents significant challenges when interpreting and using the evidence in a country such as the United Kingdom, where there are differences in both the socio‐demographic background of people experiencing homelessness, and the context within which psychosocial interventions might be accessed by them. In particular, the relatively better access to publicly funded healthcare services for people experiencing homelessness is likely to affect the experiences of individuals in the United Kingdom compared to the United States. It is worth noting, however, that there is still considerable evidence that people experiencing homelessness face significant barriers when they try to access healthcare services in the United Kingdom. These barriers are identified throughout the findings presented here, and include the geographical location of services and the ease with which services users can travel to and from these locations, eligibility criteria for accessing services, challenges when accessing mainstream services (particularly healthcare); the extent to which service users feel comfortable and invited, as well as issues of stigma and being judged.

### 6.3 Quality of the Evidence

The seven included studies were appraised for methodological limitations using Campbell's Critical Appraisal Tool for Primary Studies (White and Narayanan 2021, 60). These assessments were either undertaken by a team from the Campbell Collaboration in the development of the EGM, or for included studies that were not covered by the EGM, by two researchers involved in this review.

Overall, the seven studies demonstrate significant methodological limitations in relation to sampling and recruitment. It is not clear in many of the students how participants were recruited, nor was the relationship between researchers and participants adequately considered. The seven studies demonstrate minor limitations in relation to clarity of research questions, methods used, and appropriateness of the methods, and also in relation to data collection and data analysis.

Finally, the included studies demonstrate some methodological limitations around the conclusions drawn and recommendations made.

### 6.4 Potential Biases in the Review Process

This review was not solely based on searches undertaken by the review team. While the team did undertake hand searches of relevant journals, completed a call for evidence, and unpacked relevant systematic reviews, the primary source of studies subjected to title and abstract and full review was an existing EGM. The searches for assessments of eligibility for inclusion and risk of bias assessments were undertaken by a team from the Campbell Collaboration for the Centre for Homelessness Impact to existing and published standards.

The review has extracted data on the experiences, perceptions, views and opinions of individuals experiencing homelessness, as expressed themselves or as presented in authors' analysis of their views. The primary studies included in this review provide a curated version of the experiences of these individuals, as it is likely that only a fraction of the primary data collected is presented in each of the included studies. The reviewers have not had access to the interviews or focus groups transcripts, and are unable to comment on whether the material quoted or referenced in the included studies is an accurate reflection of the material in these transcripts. This is, of course, a limitation of all qualitative evidence syntheses, but it is worth stating as the primary data included in this report present a limited view of the data included in the primary studies, which themselves are a limited view of the overall data on the experiences and perceptions of participants involved in those primary studies.

### 6.5 Agreements and Disagreements With Other Studies or Reviews

There are no published reviews that are directly comparable to the study set out here. There are, however, three published studies that explore the experiences of people experiencing homelessness that are in part relevant to our review (Carver et al. 2020; Curry et al. 2021; Magwood et al. 2020).

Carver et al. (2020) explored the perspectives of people experiencing homelessness on what they consider important to making substance use interventions effective. The authors identified 21 relevant studies (reported in 23 papers). Three of the included papers examined psychosocial interventions, covering two studies. Rayburn and Wright (2009, 2010) both draw on a study of 10 men using a 12‐step programme in Orlando, Florida, and the first of these is an included study in our review. We also included Salem et al. (2013). As with our review, Carver et al. found that most of the relevant literature was from North America (11 studies from the United States, 7 from Canada), with three studies from the United Kingdom. Of the included studies, three focused on women participants and five on men. Methods used in the included studies included interviews, focus groups, or both. Carver et al. (2020) used a different method of qualitative synthesis to our review, namely meta‐ethnography. Because of the different focus of the Carver review to ours, the different synthesis methods used, and the limited cross over in included studies, we do not believe that the findings of our study and those of Carver et al. (2020) are comparable.

Curry et al. (2021) explored the barriers and facilitators to successful implementation of interventions aimed at addressing young people's homelessness, as identified by young people themselves and by practitioners. They included studies covering the age range of 13–25, compared to our specified age of 18 and over. The authors included qualitative, mixed, and quantitative studies in their review, and state that they used meta‐synthesis to generate findings. The Curry review identified 47 relevant studies, of which two were specifically about psychosocial interventions. One of these two (Lynch et al. 2017) is included in our review; the other (McCay et al. 2015) was not included. Although about a relevant intervention, McCay et al. was excluded as it did not include data relevant to our review (i.e., data about the experiences, perspectives or views of people experiencing homelessness). Curry et al. report that 32 of their 47 included studies were from North America (26 from the United States, and 6 from Canada); they do not report on how many studies focused on women or men only. Because of the different focus of the Curry review from ours, the different synthesis methods used, and the limited cross‐over in included studies, we do not believe that the findings of our study and those of Curry et al. (2021) are comparable.

The final review that should be considered here is that by Magwood et al. (2020) on the acceptability of health and social interventions to people experiencing homelessness. The authors published a typology of interventions as part of their inclusion criteria, none of which are relevant to our review. Magwood and colleagues identified 35 relevant studies, and there is no crossover between their included studies and those included in our review. The authors report that of the 35 included studies, 28 were from North America (14 each from the United States and Canada). The authors used ‘best fit’ framework analysis as their synthesis method. As with the Carver review and the Curry review, because of the different focus of the Magwood review from ours, the different synthesis methods used, and the lack of crossover in included studies, we do not believe that the findings of our study and those of Magwood et al. (2020) are comparable.

## 7 Authors' Conclusions

### 7.1 Implications for Practice and Policy

The seven studies included in this qualitative evidence synthesis cover a number of different psychosocial interventions. The underlying primary study included 84 individuals experiencing homelessness who were accessing these psychosocial interventions. The participants were fairly evenly split between men and women. The studies are overwhelmingly from North America, with only one being from outside of North America (from Scotland). Factors such as differences in welfare systems, the skills, roles, and approaches of staff, and access to housing, as well as differences in design and delivery between the interventions covered, need to be taken into account when considering the findings of this review. There are, however, two broad implications that we recommend policy makers and practitioners consider, as set out below.

#### 7.1.1 Put People Experiencing Homelessness at the Centre of the Design of Psychosocial Interventions

Individuals experiencing homelessness play a pivotal role in their own change journey, and the evidence generated from this review suggests that engaging individuals experiencing homelessness in key decisions about the design, delivery, and assessment of psychosocial interventions is an important part of this journey. This goes further than the ethos of the intervention or service; it means considering the physical location and layout of buildings where the intervention or service is delivered, the relationship between staff and individual service users, and recognising that individuals face stigma, discrimination, and bureaucratic barriers when trying to access other public services. It also means engaging individuals with lived experience as mentors and staff.

#### 7.1.2 Treat People Experiencing Homelessness as Individuals

People experiencing homelessness who access and use psychosocial services are not a homogenous group. They have unique and often different backgrounds and experiences, and want to be treated as individuals. Their change journeys will be unique to them, unfolding at different paces and to varying outcomes. The relationships between staff and service users are pivotal to this; positive, non‐judgemental, trusting relationships can make a positive difference for service users. The importance of staff members who themselves have first hand experience of homelessness is also key. An individual's hope and motivation is a fundamental ingredient to their change journey, and is as important as the design of the intervention or service. Maintaining this hope as individual service users deal with the ‘snakes and ladders’ of their change journey is important.

### 7.2 Implications for Research

As with any qualitative evidence synthesis, the quality of conclusions drawn and recommendations made is dependent on the breadth and rigour of the primary studies included in the review. In this case, the seven studies generally present low risk of bias, but there are only seven studies. This significantly limits the extent to which we can identify research implications. There are, however, three implications for research arising from this review.

#### 7.2.1 The Role of Hope and Motivation as a Causal Ingredient in the Effectiveness of Interventions

The qualitative evidence synthesised in this review suggests that an important ingredient to the success or not of psychosocial interventions is the hope and motivation of the individual service user. And yet, this is not an intervention component that is identified in the effectiveness studies as being a significant independent or moderating variable. Of course, measuring hope and motivation is an inherently complex task, not least because levels of hope are likely to vary over a study's duration in ways that might not be captured in effectiveness studies. However, as this appears to be an important ingredient in the seven studies we included in this review, we raise the question of whether hope and motivation might play a more pivotal role in homelessness interventions than the extant research would indicate.

#### 7.2.2 Complementariness of Effectiveness and Experiences Research

The review team had hoped to synthesise the findings of the two reviews being conducted around psychosocial interventions for people experiencing homelessness. These two reviews – effectiveness and experiences – were intended to complement each other, so as to enable evaluation of whether and how interventions might work, and how they were experienced by people with lived experience of homelessness.

It has not been possible to complete this type of synthesis. There is no direct connection between the interventions covered by effectiveness studies and those covered by qualitative studies. This lack of complementariness limits the explanatory potential across both studies and, thus, the contribution to knowledge and the policy relevance.

#### 7.2.3 Need of Increasing the Geographic Coverage of Research

This is not the first review to find a North American bias in the underlying research. Both the effectiveness review and this experiences review rely on studies from North America, particularly the United States. Primary studies from other countries are needed and would provide unique insights from the evidence base, not least as they would enable deeper understanding of, and ability of policy makers to take into consideration, the role that context plays in the effectiveness of, and experiences of, psychosocial interventions for adults experiencing homelessness.

## Author Contributions

- Content: Dr Chris O'Leary, Dr Esther Coren, Antony Roberts.
- Systematic review methods: Dr Chris O'Leary.
- Statistical analysis: Not applicable.
- Information retrieval: Anton Roberts, Dr Chris O'Leary.

## Conflicts of Interest

The authors declare no conflicts of interest.

## Supporting information

Supporting information.

Supporting information.
